# Pulmonary hypertension among 5 to 18 year old children with sickle cell anaemia in Nigeria

**DOI:** 10.1371/journal.pone.0184287

**Published:** 2017-09-14

**Authors:** Ogochukwu J. Sokunbi, Ekanem N. Ekure, Edamisan O. Temiye, Roosevelt Anyanwu, Christy A. N. Okoromah

**Affiliations:** 1 Department of Paediatrics, Lagos University Teaching Hospital, Idi-Araba, Lagos, Nigeria; 2 Department of Paediatrics, College of Medicine of the University of Lagos, Idi-Araba, Lagos, Nigeria; 3 Central Research Laboratory, College of Medicine of the University of Lagos, Idi-Araba, Lagos, Nigeria; Loyola University Chicago, UNITED STATES

## Abstract

**Background:**

Pulmonary hypertension (PHT) is a significant cause of mortality in patients with sickle cell disease (SCD). Few studies on PHT in SCD have been carried out in children. This study aimed to estimate the prevalence of PHT in children with sickle cell anaemia (SCA) and determine its clinical and laboratory correlates.

**Methods:**

In this cross sectional study, evaluation involved obtaining bio-data, history and physical examination findings in 175 SCA subjects with haemoglobin genotype SS aged 5 to 18 years and 175 age and sex matched controls with haemoglobin genotype AA. PHT was determined using peak Tricuspid Regurgitant Velocity (TRV) obtained from echocardiography as a marker. Complete blood count (CBC), lactate dehydrogenase (LDH) assay, reticulocyte count, foetal haemoglobin (HbF) estimation as well as Human Immunodeficiency Virus (HIV) I and II, Hepatitis B Virus (HBV) and Hepatitis C Virus (HCV) screening were done for patients with SCA.

**Results:**

The mean peak TRV of subjects with SCA and controls was 2.2 ± 0.4 m/s and 1.9 ± 0.3 m/s respectively and prevalence of PHT among children with SCA and controls was 22.9% and 2.3% respectively. PHT in SCA correlated negatively with body mass index, haematocrit and haemoglobin.

**Conclusion:**

This study affirms that PHT prevalence is high in children with SCA in Nigeria. Cardiovascular examination for signs of PHT is recommended for children with SCA and if required, further echocardiographic assessment from as early as five years.

## Introduction

Sickle cell disease (SCD) affects about 20–25 million individuals worldwide mostly of African, South and Central American, Caribbean, Saudi Arabian, Indian and Mediterranean ancestry.[1] It is most prevalent in sub Saharan Africa with an estimated 12–15 million cases in the region and affects two to three percent of Nigerians.[1] Morbidity from SCD may be due to anaemia, acute chest syndrome, infections, cerebrovascular accidents, pulmonary hypertension (PHT), chronic leg ulcers, retinopathy, priapism (in males) and nephropathy.[2] Common causes of death are infection, stroke, splenic sequestration, pulmonary emboli/thrombi, renal failure, pulmonary hypertension, hepatic failure, massive haemolysis, red cell aplasia and left ventricular failure.[3] During the last two decades, the management options for individuals born with SCD have improved markedly resulting in higher survival rates. As children with SCD live longer, the frequency of end-organ complications may increase [4] one of which is pulmonary hypertension (PHT).

Pulmonary hypertension (PHT) is defined as elevated mean pulmonary artery pressures measured during right sided cardiac catheterization.[5, 6] Measurement of TRV by echocardiography provides a non-invasive screening tool for the assessment of PHT and has been recommended as the initial non-invasive modality in the screening and evaluation of PHT. [7, 8] TRV is used to estimate the pulmonary artery systolic pressure using the Bernoulli equation (Pulmonary Artery Systolic Pressure = 4 (TRV)^2^ + Right Atrial Pressure) [7]. In SCD, this estimated pulmonary systolic pressure correlates well with measurements obtained by cardiac catheterization.[9] A value of 2.5 m /s or more corresponds to an estimated pulmonary artery systolic pressure of 35 mm Hg, which is approximately 2 SD above the normal mean value; for patients less than 40 years of age. [10] While some authors have defined PHT as TRV of 3.0 m/s or more, values of at least 2.5 m /s have been associated with an increased risk of death among patients with SCD. [9]

PHT is a life-threatening complication of SCD which occurs in 20% to 40% of adults with SCD and may be clinically silent until late in the course of the disease.[9] It has been recognized as a severe complication of SCD linked to accelerated mortality [9, 11] thus, PHT secondary to SCD has now been classified as a separate entity.[12] Mortality related to SCD- PHT may be as high as 40% [13] or 10-fold higher compared with those with normal TRV. [9]

There is paucity of knowledge about the prevalence of PHT in the paediatric SCD population in the developing countries and thus its significance in this age group is not well established. While there are studies in paediatric populations from developed countries which reveal a prevalence of 20 to 33% [14, 15], extrapolating these findings to the paediatric population in developing countries may be misleading. First the prevalence and severity of SCD complications varies with differences in haemoglobin haplotype which varies by region. Second the prevalence and severity of these SCD complications is also influenced by the spectrum of comprehensive medical care received. For instance children in developed countries receive routine care including chronic blood transfusion, hydroxyurea therapy and pneumococcal prophylaxis whereas only small percentages of children with SCD receive this level of care in developing countries including Nigeria. Therefore, it is hypothesized that the prevalence of SCD-PHT may be higher in children living in developing countries such as Nigeria. Hence the objectives of this study were to determine the prevalence of PHT in the paediatric sickle cell population and to identify its clinical and laboratory correlates in a cohort of Nigerian children.

## Materials and methods

Ethical approval for this study was obtained from the Health Research Ethics Committee of Lagos University Teaching Hospital Idi Araba, Lagos. Study participants were recruited from the SCD clinic of a federal government owned tertiary hospital from June to December 2014. The study questionnaire was pretested a month prior to commencement of the study by administering it to ten randomly selected patients in the SCD clinic to ascertain how user-friendly the questions were to study participants. Following this pilot, questions which seemed ambiguous to respondents were rephrased to ensure the validity of the answers and also modified as necessary to aid subject comprehension. Consecutive patients with electrophoretic documentation of sickle cell anaemia (haemoglobin SS genotype) between 5 and 18 years of age in steady state; defined as being free from acute illness for a minimum of 14 days prior to enrolment [14, 16] were included in the study while healthy age and sex matched controls who had electrophoretic documentation of haemoglobin AA genotype were recruited as controls. Only patients with haemoglobin SS (sickle cell anaemia) were included in this study due to the low prevalence of other sickle cell syndromes in this environment. Written informed consent was obtained from parents or guardians of all study participants and additional personal informed written assent from those aged ten years and above. Patients previously diagnosed with congenital heart disease, chronic renal failure, chronic liver disease, restrictive lung disease or Hepatitis B, Hepatitis C or HIV infections were excluded from the study. Full clinical examination and anthropometry was carried out on subjects and a pre-tested questionnaire (S1 Appendix) was administered to obtain biodata, history and clinical examination details.

### Echocardiography

Echocardiography was done by one of the authors (OJS) using a Sonoscape SS1–8000 Series Mobile Digital Colour Doppler Ultrasound System (Sonoscape Medical Corp, China) with a 2.0 to 5.0 MHz phased array transducer. An initial scan was done to rule out structural heart disease and subjects with normal heart scans had tricuspid regurgitant velocities (TRV) measured. Tricuspid regurgitation was assessed in three views: parasternal short-axis, parasternal long axis right ventricular inflow and apical four-chamber to determine a good quality velocity/time wave and the highest velocity obtained from any of the three views was selected as the peak TRV (m/s). PHT was defined as peak tricuspid regurgitant jet velocity of ≥2.5m/sec.

### Laboratory procedures

Complete Blood Count was done using Swelab Alpha Automated haematology machine (Boule Medical AB Stockholm, Sweden); screening for hepatitis B and C viruses using “Diaspot” test kits; HIV screening using “Determine” HIV 1 & 2 Test Kit (Alere Medical Japan) and HIV 1&2 STAT-PAK (Chembio Diagnostic New York) respectively; lactate dehydrogenase assay by turbidometry using Roche Hitachi Cobas C311 machine; haemoglobin F estimation by Betke’s Alkaline denaturation method and reticulocyte count using immunochromatography.

### Follow up of study participants

Laboratory and echocardiographic results were made available to study participants. This was done mainly by distributing personal hard copies of laboratory results to patients and their parents or guardians during follow up clinic appointments and in few cases the results were sent to patients via emails. Post—test counseling for viral infections was not necessary as none of the participants was found to be positive for any of these infections. Verbal echocardiographic reports were communicated to all patients and/ or their care givers immediately after evaluation but hard copies of echocardiographic reports were only made available to participants found to have PHT for documentation and subsequent follow up with their managing physicians. This information was also communicated to the individual physicians by issuing referral letters. Participants with PHT were counseled and advised to have follow -up echocardiographic assessment 6 months post recruitment in order to monitor progress of the complication.

### Data analysis

Statistical analysis was performed using Statistical Package for Social Sciences, (SPSS) (release 16.0.1; SPSS Inc, Chicago, IL, USA). Continuous variables were expressed as means (±SD) for normally distributed data. The distribution of continuous variables did not differ from a Gaussian (normal) distribution. Demographic, clinical findings and echocardiographic data were compared between the SCA and control groups using the student's t-test, Chi square test (or Fischer’s exact test) as appropriate for continuous and categorical data respectively. Demographic, clinical and laboratory data of SCA patients were also compared according to presence of pulmonary hypertension (TRV≥2.5m/s vs TRV < 2.5m/s) using the student's t-test for normally distributed continuous variables and the Chi square test and Fischer’s exact test for categorical variables. Pearson’s Correlation Coefficient (r) was used to assess the relationship between Tricuspid Regurgitant Velocity (TRV) and age, sex, body mass index and laboratory markers (haematocrit, haemoglobin, white blood count, platelet count, lactate dehydrogenase, reticulocyte count and haemoglobin F). Multivariate analysis by logistic regression was carried out and Odds Ratios (OR) were calculated for clinical data which were statistically significant on Chi square testing to determine independent associations of PHT. For all tests, statistical significance was defined as *p* value < 0.05.

## Results

**Fig 1:**
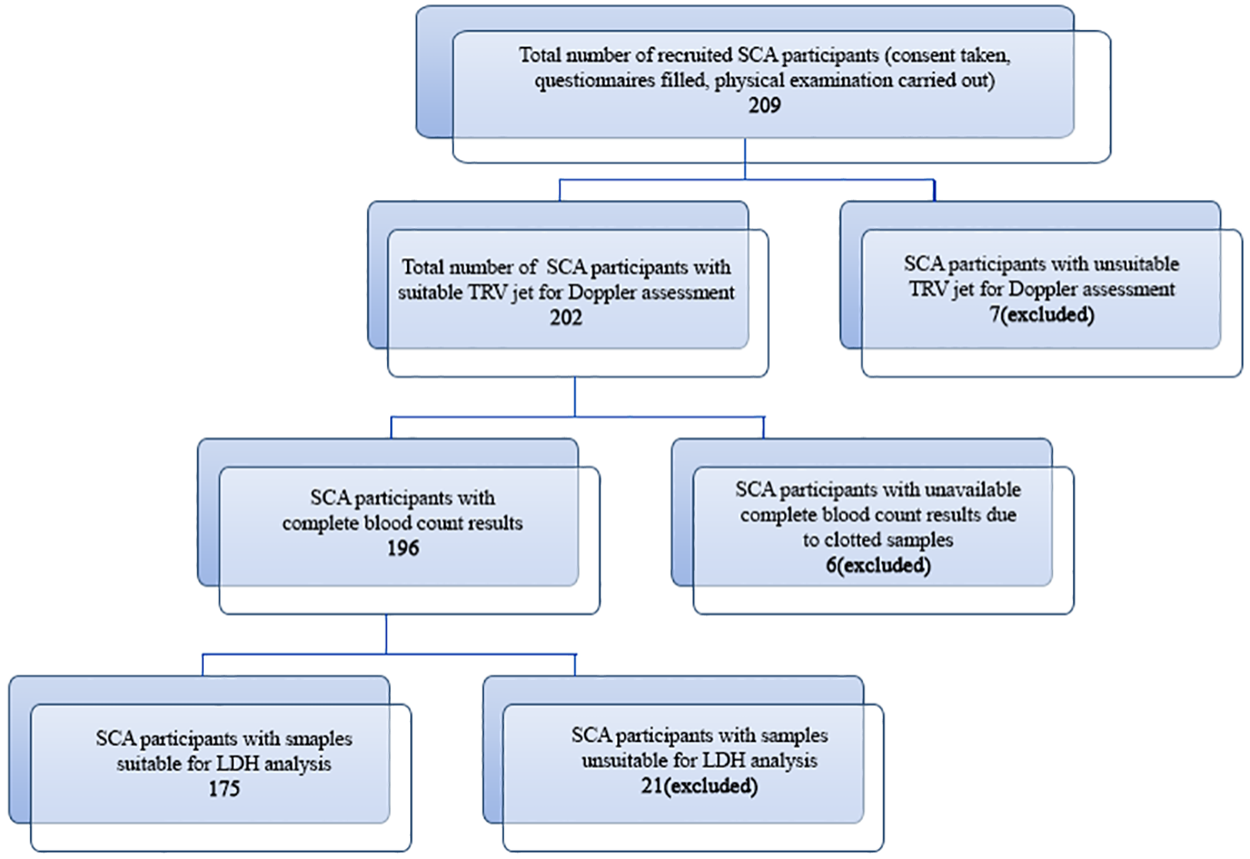
Flow chart showing recruitment of SCA subjects.

A total of 209 SCA patients were recruited for the study. Seven of them were excluded from data analysis on account of unsuitable Tricuspid regurgitation colour Doppler signals. Six subjects were also excluded on account of inability to run complete blood count test due to clotted samples and 21 subjects had unsuitable samples for LDH analysis. (Fig 1)

**Fig 2:**
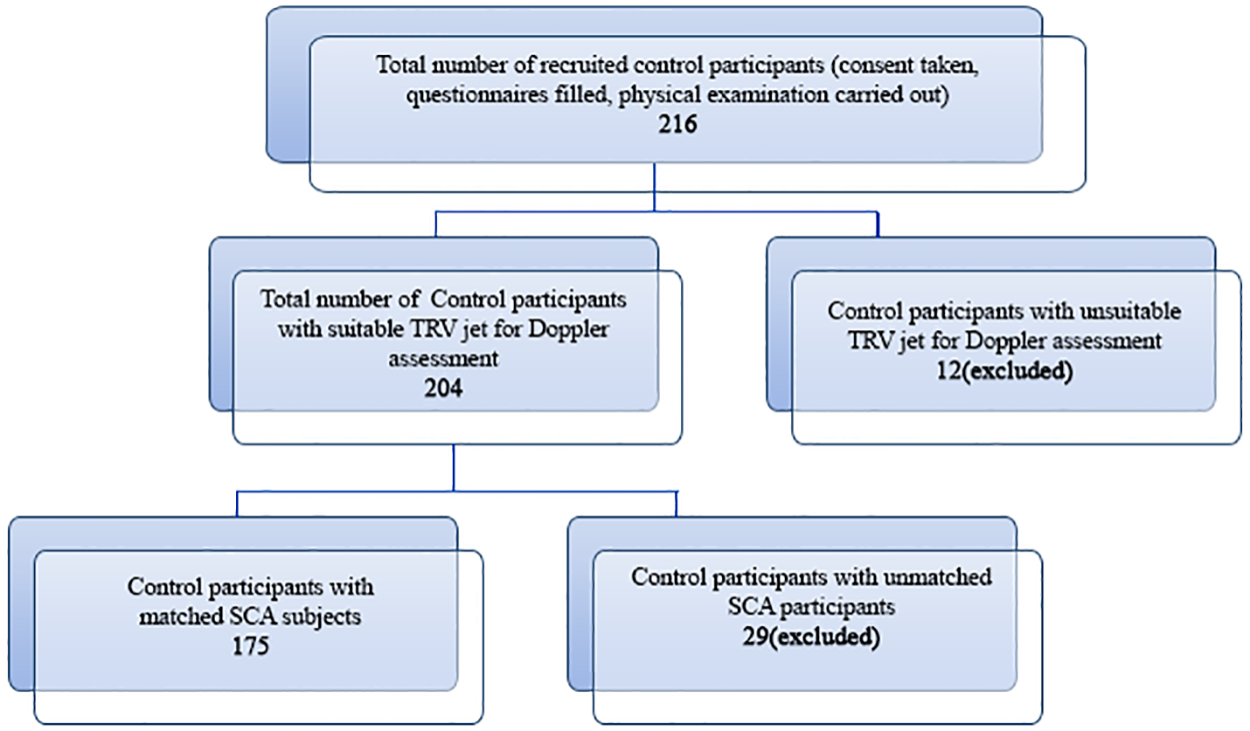
Flow chart showing recruitment of control subjects.

Among controls, 12 subjects were excluded from data analysis on account of insignificant Tricuspid regurgitation for proper wave analysis and 29 subjects were not included on account of non—availability of SCA patients to be matched with them. (Fig 2)

### Characteristics of SCA subjects and controls

A total of 350 participants were included in the data analysis for the present study: 175 SCA subjects and 175 healthy age and sex matched controls. Table 1 shows a summary of the demography and anthropometric findings in SCA subjects and controls. The mean ages of SCA subjects and controls were not significantly different (*p* = 0.937). Sixty-three percent of SCA subjects and 71.4% of controls were aged between five and 10 years, 32.3% and 21.1% of SCA subjects and controls respectively were between 10 and 15 years old while 2.9% of SCA subjects and 7.4% of controls were between 16 years and 18 years old. The male—female proportion was 54.9% to 45.1% for SCA subjects and 45.1% to 54.9% for controls respectively (*p* = 0.07). The mean height, weight and body mass index were lower for SCA subjects compared to controls while 97.1% of SCA patients and 81.2% of controls were underweight. The difference in mean oxygen saturation (%) between SCA patients and controls was statistically significant (96.9±2.5 and 97.9±1.2 respectively, *p*< 0.001).

**Table 1:** Demography and anthropometry of study population.

Characteristics	SCA subjects n = 175	Controlsn = 175	Test statistics	*p*—value
Age, mean (±SD), years	8.8±3.3	8.8±3.4	t = 0.1	0.937
Male, n (%)	96 (54.9)	79 (45.1)	χ^2^ = 3.3	0.070
Height, mean (±SD), cm	127.8±16.4	134.2±18.1	t = 3.4	**0.001**^#^
Weight, mean (±SD), kg	24.4±9.4	30.3±13.4	t = 4.8	**0.000**^#^
BMI, mean (±SD), kg/m^2^	14.5±2.1	16.1±2.4	t = 5.3	**0.000**^#^
Underweight, n (%)	170 (97.1)	142 (81.2)	χ^2^ = 23.7	**0.000**^#^

SD: standard deviation BMI: body mass index

^#^*p* value statistically significant

### Clinical findings and clinical events in SCA subjects

Clinical findings in SCA subjects were: icterus (56.6%), hepatomegaly (48.6%), loud second heart sound (20.6%), splenomegaly (13.1%), parasternal heave (12.0%) and digital clubbing (1.1%). The mean respiratory and pulse rates were 23.2 ± 5.1 cycles per minute and 94.4 ±15.1 beats per minute respectively. The average number of crisis per year was 2.67 ± 2.21 while the mean number of admissions for vaso-occlusive crisis per year was 0.84 ±1.31. The mean duration since the last episode of vaso-occlusive crisis was 6.62 months prior to participants’ recruitment for the study. Among the 175 subjects with SCA, 7.4% had a history of at least one cerebrovascular accident and 9.1% had a previous episode of acute chest syndrome. Four out of 96 male patients with SCA had a history of priapism and none of the patients reported a history of chronic leg ulcers. Among the SCA subjects, 62.4% had been transfused prior to recruitment. Nineteen out of 175 patients (10.9%) had commenced chronic transfusion and for 84.2% of them (16 of 19) this was initiated in the preceding six months. Fifty-five out of 175 SCA patients (31.4%) had commenced hydroxyurea treatment and the mean duration since onset of therapy was 14.9 ±12.3 months.

### Tricuspid regurgitant velocities

**Fig 3:**
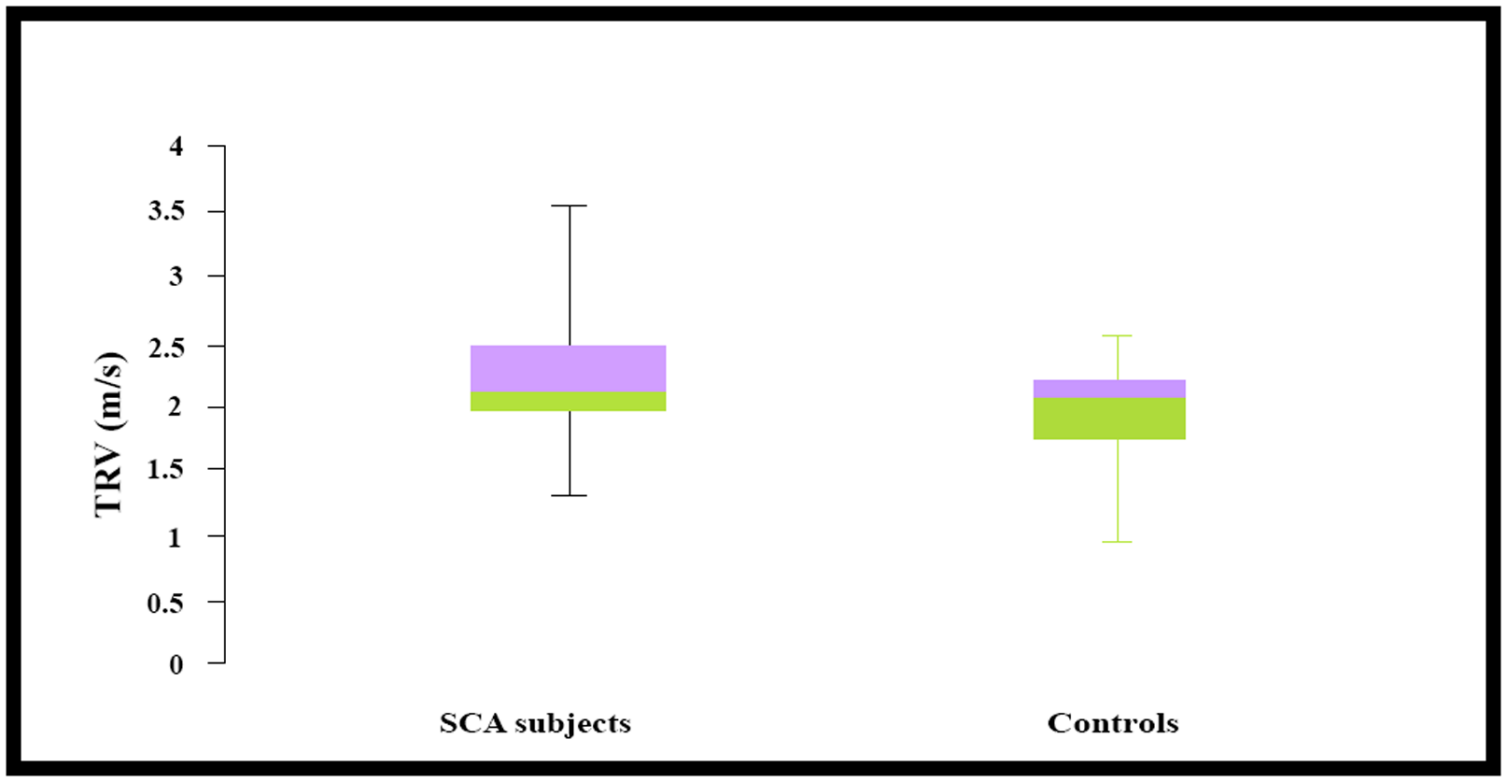
Box plots depicting TRV values among SCA subjects and controls.

The mean peak tricuspid regurgitant velocity (in m/s) among subjects with SCA was 2.2±0.4 and this was significantly higher than the mean peak TRV measured among controls which was 1.9±0.3 (t = 5.9 p< 0.001). The median and inter-quartile range of peak TRV for SCA subjects and controls are shown in Fig 3. Among SCA subjects, the range of TRV was 1.31 to 3.47 m/s and the range of TRV was 1.07 to 2.62 m/s among controls.

**Fig 4:**
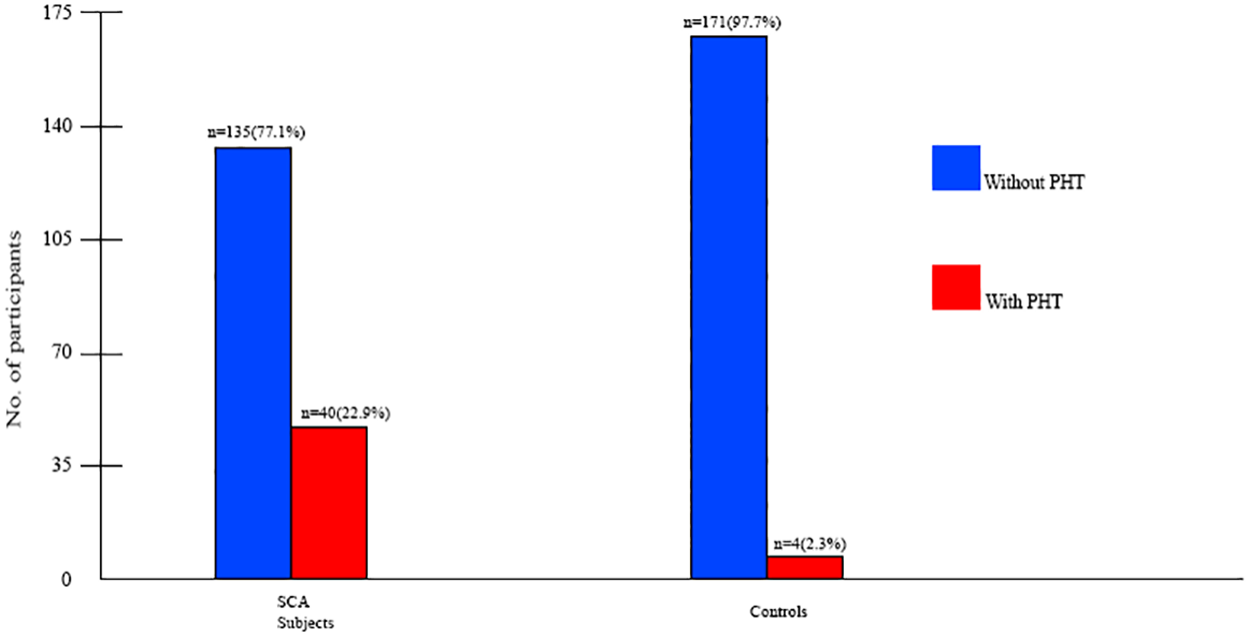
Prevalence of pulmonary hypertension in SCA subjects and controls.

The proportion of SCA subjects with PHT as defined by TRV ≥ 2.5m/s was higher than the proportion of controls with PHT (χ^2^ = 33.7 *p*< 0.001). Fig 4 shows the prevalence of PHT among SCA subjects and controls which was 22.9% and 2.3% respectively.

**Fig 5:**
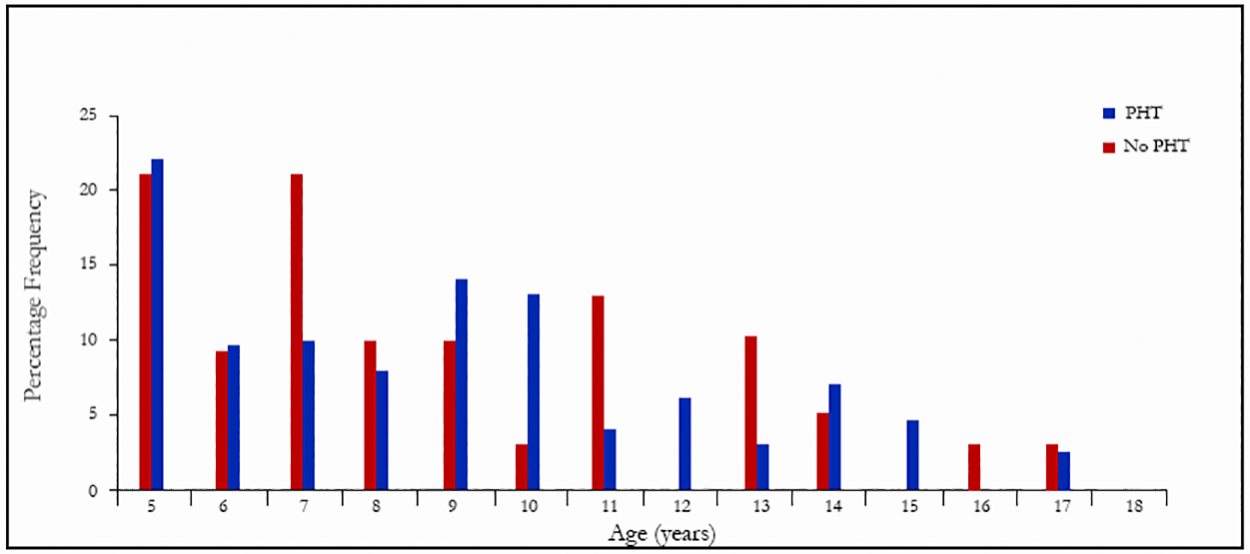
Age distribution of SCA subjects with and without PHT.

Four control subjects had PHT; two of them were aged 16 years while the other two subjects were five and eight years respectively. Fig 5 illustrates the age distribution of SCA subjects with and without PHT. The modal age was five years and the highest percentages of SCA subjects with PHT were found among those aged five and seven years respectively (20.8% in both groups). However, the younger children aged five and seven years did not differ significantly from older children in terms of history, physical and laboratory findings.

There was no significant difference between the age distributions of SCA subjects with and without PHT (χ^2^ = 1.7, *p* = 0.423).

Table 2 showed no significant difference in demographics and clinical history in SCA subjects based on the presence or absence of PHT. Age distribution was similar in both groups. Acute chest syndrome had occurred more frequently in SCA subjects without PHT but in both groups, there was a similar proportion of patients who had at least one cerebrovascular accident.

**Table 2:** Demography and clinical history of SCA subjects with and without PHT.

Characteristics	PHT n = 40	No PHT n = 135	Test statistics	*p*—value
**Demographics**				
Age, mean (±SD), years	8.8±3.5	8.8±3.3	t = 0.0	0.996
Male, n (%)	26 (65.0)	70 (51.8)	χ^2^ = 2.2	0.142
**Clinical history**				
Number of admissions for VOC in the preceding 12 months mean (±SD)	0.6±0.9	0.9±1.4	t = 1.5	0.146
Duration since last admission mean (±SD) months	15.8±16.9	15.1±27.2	t = 0.2	0.869
Duration since last crisis mean (±SD) months	8.2±11.1	6.1±10.3	t = 1.1	0.279
Previous acute chest syndrome n (%)	3 (7.5)	13 (9.6)	χ^2^ = 0.2	0.681**
Previous cerebrovascular accident n (%)	3 (7.5)	10 (7.4)	χ^2^ = 0.0	0.607**
Previous priapism, males only n (%)	2 (5.0)	2 (1.5)	χ^2^ = 1.7	0.225**
Chronic transfusion therapy n (%)	4 (10.0)	15 (11.1)	χ^2^ = 0.0	0.553**
Duration of chronic transfusion therapy mean (±SD) months	7.3±3.2	6.1±2.9	t = 0.7	0.482
Hydroxyurea therapy n (%)	13 (32.5)	42 (31.1)	χ^2^ = 0.03	0.868
Duration of hydroxyurea therapy mean (±SD) months	12.0±10.7	15.8±12.7	t = 0.9	0.339

**p values obtained by Fischer exact test, SD: standard deviation

Table 3 compares the clinical findings in SCA patients with and without PHT. The presence of jaundice, parasternal heave, hepatomegaly and a loud component of the second heart sound differed significantly between SCA subjects with and without PHT. Hepatomegaly, parasternal heave and loud second heart sound were noted more commonly among SCA subjects with PHT.

**Table 3:** Clinical findings of SCA subjects with and without PHT.

Clinical findings	PHT n = 40	No PHT n = 135	Test statistics	*p*–value
Height mean (±SD), cm	129.4±17.7	131.2±17.5	t = 0.1	0.921
Weight mean (±SD), kg	24.7±9.1	27.7±12.3	t = 0.2	0.807
BMI mean (±SD), kgm^-2^	14.3±1.9	15.4±3.0	t = 0.7	0.485
Underweight n (%)	40 (100.0)	130 (96.3)	χ^2^ = 1.5	0.467
Pallor n (%)	40 (100.0)	135 (100.0)	χ^2^ = 0.0	1.000
Jaundice n (%)	11 (27.5)	65 (48.2)	χ^2^ = 5.4	**0.020**^#^
Digital clubbing n (%)	1 (2.5)	1 (0.7)	χ^2^ = 3.7	0.159
Parasternal heave n (%)	11 (27.5)	10 (7.4)	χ^2^ = 14.5	**0.002**^#^
Loud second heart sound n (%)	22 (55.0)	18 (13.3)	χ^2^ = 18.9	**0.000**^#^
Haemic murmur n (%)	12 (30.0)	33 (24.4)	χ^2^ = 3.6	0.164
Splenomegaly n (%)	7 (17.5)	16 (11.8)	χ^2^ = 0.9	0.353
Hepatomegaly n (%)	26 (65.0)	59 (43.7)	χ^2^ = 5.6	**0.018**^#^

^**#**^*p* value statistically significant SD: standard deviation BMI: body mass index

No subject was positive for Hepatitis B and C viruses and Human Immunodeficiency Virus I and II. The mean haemoglobin and haematocrit were 7.9±1.3 g/dl and 22.8 ± 3.8% respectively, mean white blood cell count was 12.7 ±7.8 x 10^9^/L and the mean platelet count was 352.9 ± 138.9 x 10^9^/L. The mean reticulocyte count, lactate dehydrogenase and haemoglobin F levels were 2.8 ± 2.9%, 724.5 ± 319.9 u/L and 6.8 ± 3.3% respectively. The laboratory characteristics of SCA subjects with and without pulmonary hypertension are shown in Table 4. SCA subjects with PHT had lower mean values of haemoglobin, haematocrit, lactate dehydrogenase, white blood cell, platelet and reticulocyte counts compared to children without PHT. Mean foetal haemoglobin was higher among SCA patients with PHT. However, none of these values were statistically significant.

**Table 4:** Comparison of laboratory parameters of SCA subjects with and without PHT.

Laboratory Characteristics	PHT n = 40	No PHT n = 135	t- test statistics	*p*—value
Haemoglobin mean ± SD (g/dl)	7.7±1.1	7.9±1.3	1.2	0.225
Haematocrit mean ± SD (%)	22.1±3.1	22.9±15.7	1.3	0.186
White cell count mean ± SD (x 10 ^9^ /L)	12.1±5.1	12.9±8.5	0.6	0.576
Platelet count mean ± SD (x 10 ^9^ /L)	333.6±155.6	357.1±133.9	0.7	0.461
Reticulocyte count mean ± SD (%)	2.5±2.4	2.8±3.1	0.5	0.605
Lactate dehydrogenase mean ± SD (u/L)	641.9±383.5	748.9±295.8	1.9	0.063
Foetal haemoglobin mean ± SD (%)	7.6±3.8	6.5±3.1	1.9	0.053

### Correlates of pulmonary hypertension in SCA

Physical findings which differed significantly between SCA subjects with and without PHT were jaundice, parasternal heave, hepatomegaly and presence of a loud component of the second heart sound. In order to control for confounders, multivariate analysis by logistic regression was carried out on these variables to identify independent associations of PHT in SCA subjects. The independently associated variables and their Odds Ratios are shown in Table 5. The presence of a loud second heart sound was a statistically significant association of PHT in children with SCA and subjects with this clinical finding were 3.4 times more likely to have PHT.

**Table 5:** Independent variables associated with PHT.

Clinical findings	Odds Ratio	95% Confidence Interval	*p*-value
Hepatomegaly	2.00	0.90–4.45	0.091
Jaundice	1.96	0.86–4.51	0.111
Loud second heart sound	3.39	1.36–8.50	0.009
Parasternal heave	3.09	0.39–24.29	0.283

Pearson’s correlation was carried out to demonstrate relationships between TRV and several factors. There was no correlation between TRV and age, oxygen saturation, number of admissions for vaso-occlusive crisis, lactate dehydrogenase, foetal haemoglobin, white blood cell, platelet and reticulocyte counts. There was significant negative correlation between TRV and body mass index, haemoglobin and haematocrit (r = -0.2, *p* = 0.001, r = -0.2, *p* = 0.021 and r = -0.2, *p* = 0.008 respectively)

## Discussion

The absolute mean TRV values found in the current study for SCA subjects and controls are comparable to figures earlier reported from Northern Nigeria (2.1 ± 0.6 and 1.8 ± 0.5 m/s respectively). [17] In comparison, SCD subjects had significantly higher values than controls. In a Western Nigeria study, the authors did not report PHT in any of their controls and there was also a significant difference between the mean peak TRV of the controls and that of SCD patients. [18] This significant difference in TRV values between SCD patients and controls as well as the very low prevalence of PHT among control subjects suggests a contributory effect of the haemoglobin genotype on the degree of tricuspid regurgitation velocity.

The prevalence of PHT among children with SCD in this study was 22.9% which was similar to figures reported by earlier investigators. [19, 20] Two Nigerian studies which included children and adults found a wide prevalence of 3.6% to 25%. [17, 18] The Western Nigeria study [18] with prevalence of 3.6% had a small sample size which was about one-third that of the current study and this may have accounted for the low prevalence of PHT found in their cohort. These investigators also proposed that their cohort was highly selective and better motivated for regular care compared to the Northern Nigerian participants.

The similar prevalence documented in the current study compared to data from international studies in SCD patients of diverse descent and age range supports reports that this complication is neither race nor age dependent. Some of these cohorts have addressed this problem with study populations that cut across childhood to adulthood and others have studied patients of other races, but this study was carried out in a strictly paediatric population in Sub Saharan Africa. These findings highlight the importance of this complication in a sub—region that accommodates about 60% of the world’s SCD population. [1]

Elevated TRV was found in 2.3% of controls in the present study population which was lower than 7% noted in a Northern Nigerian study [17] but was higher than the Lagos, Nigeria study in which none of the controls had elevated TRV. [18] The control group in the Lagos study involved only patients with genotype AA as in the current study but the Northern Nigerian study involved patients with documented haemoglobin AA as well as AS genotypes. The contributory role of the sickle cell trait in the occurrence of PHT remains to be elucidated.

In the present study, neither difference in age nor gender was significant between SCD patients with and without PHT and neither did age correlate with TRV. These findings were in keeping with similar paediatric studies [19, 21] conducted in the United States of America. The highest frequency of SCD patients with PHT was among children aged five and seven years and this trend suggests that this complication sets in quite early in life (as early as five years of age in this cohort). High prevalence of PHT among younger children is an unusual finding as this complication is thought to progress with age. Children in the modal age for PHT in this study did not show any significant difference in their laboratory parameters compared to children in other age groups. It is likely that this high prevalence is a reflection of the modal age group of patients who attend the paediatric SCD clinic of the study centre. Correlations of age with PHT in SCD have mostly been found in adult populations and are thus not comparable to the present study. [11, 20] Nonetheless, follow up of the present cohort may support this trend.

Majority of participants in both groups (SCD patients and controls) were noted to be underweight. The nutritional status of the study population could be a reflection of the population seeking for care at the government tertiary hospital. Although the study centre is a teaching hospital, majority of its patients live in the environs which include a slum part of the state or are patients in the middle income social strata as the cost of care in this tertiary facility is lower than what is offered in most private institutions providing specialist care in the state. Although TRV correlated weakly with BMI in the present study similar to observation by another author, [22] BMI did not differ significantly between the PHT and non-PHT groups.

The current study revealed no significant association between PHT and the frequency of vaso-occlusive crisis and acute chest syndrome as reported in previous works. [17, 19, 21] Conversely, in some studies [12, 23] patients with PHT had more frequent history of leg ulcers, avascular necrosis, seizures, and cerebrovascular accidents compared to patients without PHT. These complications have previously been associated with the degree of vasculopathy in SCD patients but the poor association between these vasculopathy related clinical events and the occurrence of PHT in this cohort does not support their common haemolytic etiology which has been reported previously. [21] Some of these complications are more common in adults with SCD and it is likely that the low prevalence of history of cerebrovascular accidents, acute chest syndrome and priapism in the study population affected the ability to obtain statistical significance between these clinical events and the presence of PHT in our study participants.

The current study did not reveal any relationship between the use of hydroxyurea or chronic transfusion and the occurrence of PHT, although these have been recommended as supportive therapies in the management of PHT. [24] The absence of the association of hydroxyurea use and chronic transfusion therapy with lower incidence of PHT in the current study is likely due to the relatively short duration of these therapies among our study participants. The mean duration of chronic transfusion was about 6 months while hydroxyurea had been used for a mean duration of 14 months in our cohort. There was also an insignificant trend of higher HbF levels among SCD patients with PHT compared to patients without PHT as opposed to findings in other studies. [25, 26] HbF functions by inhibiting polymerization of HbS thus reducing the frequency of vasculopathic complications like PHT. Hydroxyurea increases HbF concentrations in SCD patients but data has shown that substantially improved life spans occur following prolonged therapy for as long as six years [27] which is much higher than the mean duration of hydroxyurea use of 14.9 months found in our study. Furthermore, the cutoff level of HbF concentration for defining lower risk of severe pulmonary complications has been estimated at 20% [28] which is far above the mean HbF level of 6.8% found in our SCD cohort.

Laboratory findings from this study were in keeping with a similar paediatric and adolescent study carried out by Liem *et al* [21] in which no significant relationship was observed between TRV and percent foetal haemoglobin (Hb F), total white blood cell count (WBC) or platelet count. Similarly, in their adult cohort, Gladwin *et al* [9] also reported that the HbF level, WBC, and platelet count were unrelated to the occurrence of PHT.

Our study showed a significant, positive correlation between TRV and LDH and TRV and reticulocyte count. Several studies have documented correlation between TRV and higher LDH levels; [13, 23, 29] and others have emphasized the important role of haemolysis as a significant contributor to TRV elevation. [30, 31] These inferences have been drawn from findings of decreased haemoglobin and haematocrit with an associated increase in markers of haemolysis such as LDH and reticulocyte count. Therapies aimed at decreasing haemolysis have been postulated as measures to prevent occurrence of and delay progression of PHT in children with SCD. [21]

In the current study, the correlation between TRV and haemoglobin and TRV and haematocrit though weak, was statistically significant and similar to observations made by some other investigators. [17, 19, 21] The significant relationship observed between the occurrence of elevated TRV and reduced haemoglobin without concomitant elevation in markers of haemolysis in our cohort suggests that anaemia may contribute significantly to the occurrence of PHT in SCD patients. Some investigators have pointed out that TRV is likely enhanced in severely anemic patients with increased cardiac output and high stroke volume. [16, 22] However, the patients recruited for this study were in steady state and not observed to be severely anemic. The role of optimized haematocrit in the occurrence and/or progression of PHT in SCD patients deserve further investigation.

This study has shown a high prevalence of PHT in one of the largest SCD cohorts involving an exclusively paediatric population in the sub-Saharan African region and has addressed some of the hypotheses put forward in some previous studies about the associations and correlates of PHT in SCD.

The present study had some limitations which included: the recruitment of only patients in steady state which may have been a source of selection bias as it excluded the important subset of patients with symptomatic PHT. Information on clinical outcomes was largely obtained directly from the care givers of patients. This is because many SCD patients who are on follow up in tertiary hospitals such as the study center also receive care in peripheral hospitals during periods of acute illness. Due to the non- availability of a unified record system, these events are not documented in the clinical records of these tertiary hospitals. In this study, PHT was evaluated using echocardiography instead of cardiac catheterization which is the “gold standard” for diagnosing pulmonary hypertension. It is likely that the figures may be different if PHT was evaluated using cardiac catheterization. The nature of the study allows for demonstration of associations, but cannot infer cause-effect relationships.

## Conclusion

From the findings of the study, we recommend early and regular screening for PHT as part of routine management of children with sickle cell disorder from as early as five years of age to facilitate early diagnosis and institute therapy where necessary. Complete cardiovascular examination of SCD patients during routine follow up visits is necessary to detect abnormal physical findings such as a loud second heart sound which should prompt referral for echocardiography.

Further research should demonstrate the correlation of echocardiography derived markers of PHT with values obtained at cardiac catheterization in SCD patients in Sub Saharan Africa. This would help to generate cut off values for decision on patient referral for this expensive and invasive procedure. Also, a study of patients younger than five years of age with SCD is needed to investigate how early PHT develops while prospective studies to characterize the long term effects of, as well as risk of mortality from pulmonary hypertension in the paediatric population is required. The influence of therapy such as chronic transfusion and hydroxyurea on PHT in children with SCD also deserves further investigation.

## Supporting information

S1 Appendix Study questionnaire. (DOCX)

S2 Appendix Study data. (XLSX)
