# Supplementary material for: Pulmonary hypertension among 5 to 18 year old children with sickle cell anaemia in Nigeria
Source: PLoS One. 2017 Sep 14;12(9):e0184287. doi: 10.1371/journal.pone.0184287 (PMC5598958; doi:10.1371/journal.pone.0184287)
Supplement: S1 Appendix — (DOCX) [file pone.0184287.s001.docx]

**STUDY PROFORMA**

**BIODATA**

1. Code number:...................................................
2. Age: :...................................................
3. Sex: a) Male b) Female
4. Hospital number: :...................................................
5. Phone number: :...................................................
6. Haemoglobin genotype: :...................................................

**HISTORY**

1. Average number of crisis per year................................
2. No of hospital admissions for Vaso Occlusive Crisis in the last one year:...................
3. Date of last admission:......................................
4. Date of last crisis:................................
5. Previous Acute Chest Syndrome: a) Yes b) No

If yes, how many......

1. Previous stroke: a) Yes b) No

If yes, how many.................

1. Previous priapism: a) Yes b) No

If yes, how many..................

1. Chronic leg ulcers a) Yes b) No

If yes, for how long.......................

1. Chronic transfusion therapy a) Yes b) No

If yes, how long and how often.....................................................

1. Date of last transfusion: .............................................
2. Hydoxy urea therapy: a) Yes b) No

If yes, for how long..................................

**EXAMINATION**

1. Height....................... Weight................ BMI.................
2. Palor jaundice digital clubbing cyanosis peripheral edema
3. Pulse rate.......... Blood Pressure............Apex beat.............. Heart sounds........loud P_2.........._
4. Respiratory rate.............. oxygen saturation............................
5. Liver size..................................... Splenic size:...................................

**LABORATORY DATA**

1. Haemoglobin concentration................ Total white blood cell count................. Platelet count.................. Reticulocyte count...............
2. Lactate Dehydrogenase..............................
3. Haemoglobin F concentration.......................

**ECHOCARDIOGRAPHY DATA**

1. Tricuspid Regurgitant Velocity:............................................
2. Echocardiographic diagnosis: .........................................................................................
